# Associations between dietary diversity and self-rated health in a transverse study of four local food systems (French Guiana, Guadeloupe, Portugal and Senegal)

**DOI:** 10.1186/s12889-025-21872-8

**Published:** 2025-02-28

**Authors:** Michael Rapinski, Richard Raymond, Damien Davy, Jean-Philippe Bedell, Abdou Ka, Jean Lubszynski, Pascal Jean Lopez, Eduardo Ferreira Da Silva, Nathalie El Deghel, Enguerran Macia, Priscilla Duboz

**Affiliations:** 1 UMR 7206 Eco-Anthropologie, MNHN / CNRS Université Paris Cité, Paris, 75116 France; 2 UAR 3456 LEEISA, CNRS, Université de Guyane, IFFREMER, Cayenne, 97300 French Guiana; 3 Université Claude Bernard Lyon 1, LEHNA UMR 5023, CNRS, ENTPE, Vaulx-en-Velin, F-69518 France; 4 IRL 3189 ESS, UCAD / CNRS / UGB / USTTB / CNRST, Faculté de médecine de l’UCAD, Dakar, Sénégal; 5 UMR 8067 BOREA, MNHN / CNRS / Sorbonne Université / IRD Université de Caen Normandie / Université des Antilles, Paris, 75005 France; 6 Departamento de Geociências, Universidade de Aveiro, Campus de Santiago, Aveiro, 3810-193 Portugal; 7 Faculté de médecine Nord, UMR 7268 ADES, CNRS / EFS / AMU, Marseille cedex 15, 13916 France

**Keywords:** Health, Self-rated health, Dietary diversity score, Africa, Americas, Europe, Food systems

## Abstract

**Background:**

The nutrition transition is linked to the double-burden of malnutrition worldwide, and its impact on the quality of life is considerable. The dietary diversity score and self-rated health are two proxies that have been used to assess, for the former, nutrient adequacy and overall diet quality, and for the latter, health from a sociological, epidemiological and economical lens. The general aim of this study was to evaluate the relation between food and subjective health, and to test the hypothesis that greater dietary diversity is positively associated with a better perception of health.

**Methods:**

A transverse comparison of foods consumed in four highly contrasted local socio-ecosystems (i.e., two French oversea territories: French Guiana, Guadeloupe, Portugal and Senegal) was conducted using 24-hour dietary recalls. Dietary diversity was calculated using 18 food groups based on classifications provided by WHO and FAO. Binary logistic regressions were used to assess the relationship between dietary diversity scores and answers to the question assessing self-rated health.

**Results:**

Overall, 465 individuals, 18 years and older, from Senegal, Guiana, Guadeloupe and Portugal were interviewed using a 24-hour dietary recall. Participants were selected via a combination of non-probability sampling methods. The mean dietary diversity score for all regions combined was 9.22. Over one-third of participants reported their health as ‘good’ (39.8%), whereas ‘bad’ and ‘excellent’ health were the least reported, at 6.45% and 9.03%, respectively. Multiple binary logistic regression notably found that dietary diversity score (OR = 0.88, 95% CI [0.79, 0.99], *p* = 0.010) and at-home meal preparation, specifically with the reference category ‘all the time’ compared to ‘never’ (OR = 3.31, 95% CI [1.55, 7.07], *p* = 0.002) were statistically significant predictors of self-rated health (i.e., declaring overall bad health).

**Conclusions:**

This study demonstrates a positive association between dietary diversity and self-rated health across distinct cultural contexts. The findings reinforce the importance of diverse diets for subjective well-being, regardless of differences in food systems. Public health messaging should continue to promote dietary diversity and home-cooked meals as effective strategies for improving health. Self-rated health could serve as a useful tool for quickly assessing the outcomes of nutrition therapy.

**Supplementary Information:**

The online version contains supplementary material available at 10.1186/s12889-025-21872-8.

## Background

The nutrition transition is linked to the double-burden of malnutrition worldwide [1], and its impact on the quality of life is considerable. Indeed, it was found that improving diet could potentially prevent one in every five deaths worldwide [2]. It was also estimated that roughly 24% of total deaths worldwide in 2017 (i.e., 11 million premature deaths) were attributed to unhealthy diets linked with the development of several chronic non-communicable diseases [3]. This represents over 2.8 million deaths annually that are related to being obese or overweight alone [4]. On the other hand, it was estimated that undernutrition (i.e., underweight, wasting and stunting) caused nearly 1.5 million childhood deaths in 2016 [5]. Data from 2006 to 2018 reveal an overall prevalence of 6.3%, 13.7% and 29.1% for child wasting, underweight and stunting, respectively, in low- and middle-income countries [6].

The notion of nutrition transition, originally developed by Popkin [7] to describe five broad dietary patterns, is often employed more broadly to describe nutritional changes from a predominance of local “traditional” foods to industrialized, marketed and ultra-processed foods [8, 9]. The latter food typologies are characterized by diets higher in fats, saturated fats, and sugars, which have been linked with a higher prevalence of non-communicable diseases like obesity and type 2 diabetes [10]. This aligns with Popkin’s fourth pattern of the nutrition transition: degenerative diseases [7]. In keeping with this, dietary interventions through nutrition therapy are important to manage such illnesses and are supported by organizations worldwide [11–16]. Food and diet play important roles in overall health, and the cultural dimensions of specific food typologies also contribute to a sense of overall well-being [17–23].

Dietary diversity and self-rated health (SRH) are two proxies that have been used to assess, for the former, nutrient adequacy and overall diet quality [24], and for the latter, health from a sociological, epidemiological and economical lens [25]. Numerous studies have associated dietary diversity scores (DDS) with nutrient intake adequacy [26–31], particularly with micronutrients [32]. These scores have also been associated with biological and anthropometric indicators of nutritional status, such as height-for-age (HAZ), weight-for-age (WAZ), BMI-for-age (BAZ), BMI, weight, and height [28, 29, 33, 34]. They have also been associated with the incidence of chronic diseases like diabetes [35], obesity [36–38], cardiovascular risk factors [39, 40] and metabolomic syndrome [41], despite some systematic reviews and meta-analyses cautioning that such relationships depend on the method for determining DDS [24, 42].

Whereas DDS act as indicators to capture health from the lens of nutrition, the general outlook on health that SRH captures is articulated by: “an individual and subjective conception that is related to the strongest biological indicator, death, constitutes a cross-road between the social world and psychological experiences on the one hand, and the biological world, on the other” [25]. As one of the most widely used measures of general health in population health research, SRH is a subjective perception of individual health [43–45]. It is “embodied by an individual’s instantaneous subjective evaluation of his or her own health status. It is assumed to capture numerous aspects of one’s health status” [46].

Indeed, self-rated health is a predictive factor for various diseases and conditions, notably cardiovascular disease [47] and, in the medium and long term, a predictor of morbidity and mortality [48–51]. The World Health Survey and tests of the validity of SRH across cultures confirm that the instrument is as valid as any other measure of health status [52–56]. SRH is higher in developed countries (e.g., 70–75% of participants reported “very good” and “good” health in Europe [57], and 87% reported good to excellent health in the United States [58]) compared to Africa [46], even if information on self-rated health is rudimentary in sub-Saharan Africa. This appears logical since globally, life expectancy at birth and adult mortality rate tend to be lowest and highest, respectively, in countries from Africa [59, 60].

As expected, several factors influence SRH that can be summarized through socio-demographic, socio-economic, psychological and sanitary characteristics [61]. These also correspond to many factors that explain the evolution of food systems in which nutrition and dietary transitions occur, most notably through the influence of agro-industrial foods from an increasingly globalized food system [8, 62]. Indeed, there is overwhelming evidence that links the consumption of energy-dense and nutrient-poor foods, characteristics of ultra-processing, with an increased risk of developing chronic and metabolic diseases [63–70]. But there is also a growing body of evidence that suggests how individuals’ perceptions of their health are related to numerous health outcomes that include type 2 diabetes [45, 71, 72], elevated inflammatory markers [73, 74], obesity and overweight [75], genetic factors [76], and lifestyle factors like physical activity, smoking, alcohol consumption and unhealthy meal planning choices [77].

More recently, studies have focused on eating behaviors and practices. Positive eating practices, like preparing meals at home, show that time spent on meal preparation is linked to better self-rated mental health and stress [78, 79], whereas SRH increased with the number of homemade meal preparation episodes [80]. Among Chinese elderly, An et al. [81] found that the daily consumption of certain types of food, i.e., fruit, meat, fish, egg, and tofu, improved SRH, whereas the daily consumption of sugar was associated with a poorer assessment. Furthermore, food insecurity was generally associated with poorer SRH [82, 83]. Despite these studies highlighting the link between food and individuals’ perception of health, none, to the best of our knowledge, have explicitly shown the relationship between these two simple and easy-to-use scores for assessing subjective health (i.e., SRH) and objective health through diet (i.e., DDS).

Hence, the general aim of this study was to evaluate in greater depth the relation between food and subjective health in a transverse comparison of foods consumed in four highly contrasted local socio-ecosystems from three different countries (i.e., Portugal, Senegal, and two overseas departments of France: French Guiana and Guadeloupe). These four socio-ecosystems can be characterized by different phases of the nutritional transition [62]. Some areas of the Sahel in Senegal recovering from the consequences of the 1970s drought still bear the characteristics of the pattern of receding famine [7, 62]. In the French overseas territories (Guadeloupe and French Guiana), the prevalence of chronic non-communicable diseases, namely diabetes, overweight and obesity, testifies to Popkin’s fourth nutritional transition model (i.e., degenerative diseases) [7, 62]. Finally, in Portugal, data on diet and health suggest that the nutrition transition has stabilized [7, 62]. Food groups declared in 24 h dietary recalls were therefore assessed to test the hypothesis that greater dietary diversity is positively associated with a better perception of health. To evaluate what other factors are essential modulators of self-rated health, socio-demographic and socio-economic characteristics were also assessed.

## Methods

### Study sites

**Fig. 1:**
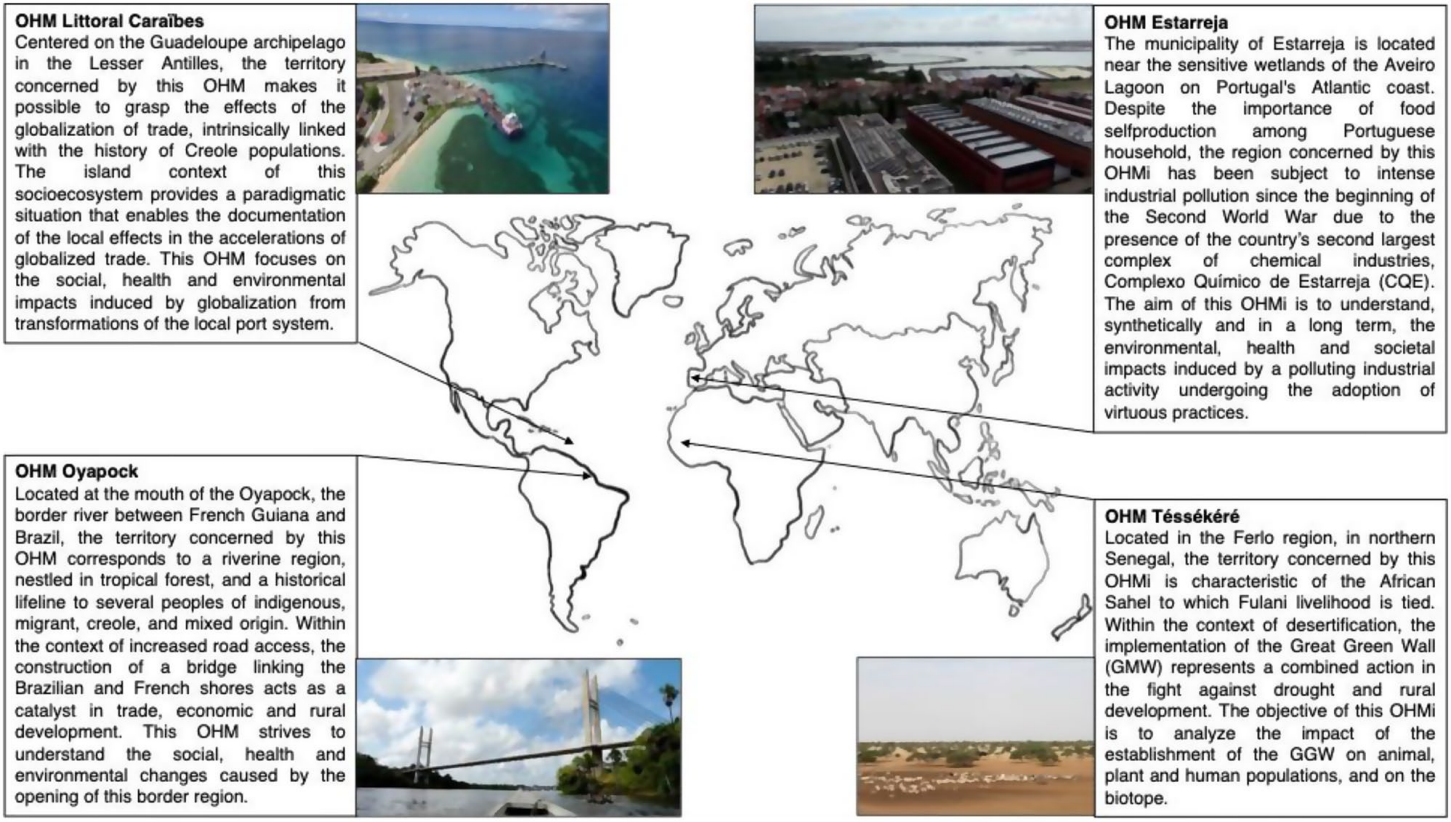
Description of the regions impacted by the disrupting event identified by each OHM, i.e., the focal object [26]. Figure adapted from Rapinski et al. [62]

This cross-sectional study was conducted in 2023 within the framework of a survey of the evolution of local food systems that consists of a two-part assessment of individual eating habits and representations of the notion of “eating well” in four regions concerned by the research activities of four Human-Environment Observatories (OHM) [62]. These include OHMi-Tessekere (Senegal), OHMi-Estarreja (Portugal), OHM-Littoral Caraïbe, (Guadeloupe, French West Indies) and OHM-Oyapock (French Guiana), hereafter referred to as Tessekere (Senegal), Estarreja (Portugal), Caribbean Coast (Guadeloupe) and Oyapock (French Guiana), respectively (Fig. 1). A detailed explanation of these research devices, implemented as part of the French government’s “Investing in the Future Program” (*Programme d’investissement d’avenir*), and the structure within which they operate can be found in Rapinski et al. [62] and Chenorkian [84]. Briefly, “their role is to enable the study of anthropized ecosystems susceptible to socio-ecosystemic crises” [84]. Participating OHMs had to be willing to follow a standardized and similar data collection protocol.

### Data collection

Altogether, 465 individuals (Table 1), 18 years and older, were interviewed using a 24-hour dietary recall. Participants were selected, via a combination of non-probability sampling methods that included: (a) convenience sampling through haphazard and chance meetings, (b) quota sampling to ensure equitable sex ratios and the inclusion of members of different subpopulations (e.g., Creole, Indigenous, and Brazilian populations on the Oyapock River), and (c) snowball sampling either through relationships between participants and various networks.

The work and interviews were conducted:

- OHMi Tessekere (Senegal), from 26/05/2023 to 20/07/2023.
- OHMi Estarreja (Portugal), from 23/03/2023 to 15/10/2023.
- OHM Littoral-Caraïbe (Guadeloupe), from 19/03/2023 to 27/09/2023.
- and OHM Oyapock (French Guiana), from 04/04/2023 to 21/06/2023.

**Table 1:** Demographic and socioeconomic characteristics of participants in the SASI survey. Medians describe age and household size with interquartile ranges (IQR) expressing dispersions between the first and third quartiles (Q1-Q3), whereas frequencies (*n*) and percentages (%) describe other variables

	Téssékéré	Guadeloupe	Oyapock	Estarreja	Total
Sample size *N*	101	260	50	54	465
Median age (IQR)*	42 (30–52)^b^	51 (32–63)^a^	41 (30–51)^b^	51 (35–66)^ab^	47 (31–62)
Sex *n* (%)					
* Men*	41 (40.59)	110 (42.31)	25 (50.00)	15 (27.78)	191 (41.08)
* Women*	60 (59.41)	150 (57.69)	25 (50.00)	39 (72.22)	274 (58.92)
* Ratio (M: W)*	0.68	0.73	1.00	0.38	0.70
Median household size^1^ (IQR)*	8 (6–12)^a^	2 (2–3)^c^	4 (3–8)^b^	3 (2–4)^c^	3 (2–5)
Marital status *n* (%)					
* Conjugal union (all partnership type)*	92 (91.09)	111 (42.69)	28 (56.00)	27 (51.92)	258 (55.72)
* Single (incl. divorced and separated)*	6 (5.94)	138 (53.08)	20 (40.00)	21 (40.38)	185 (39.96)
* Widow*	3 (2.97)	11 (4.23)	4 (4.00)	4 (7.69)	20 (4.32)
Meal preparation in the home *n* (%)					
* Very frequently/all the time*	47 (46.53)	154 (59.23)	28 (56.00)	48 (88.89)	277 (59.57)
* From time to time*	21 (20.79)	81 (31.15)	21 (42.00)	4 (7.41)	127 (27.31)
* Never*	33 (32.67)	25 (9.62)	1 (2.00)	2 (3.70)	61 (13.12)
Meal consumption outside the home *n* (%)	18 (17.82)	105 (40.38)	13 (26.00)	15 (27.78)	151 (32.47)
Self-rated health *n* (%)					
* Poor*	23 (22.77)	3 (1.16)	0 (0.00)	4 (9.76)	30 (6.67)
* Passable*	38 (37.62)	56 (21.71)	15 (30.00)	8 (19.51)	117 (26.00)
* Good*	17 (16.83)	126 (48.84)	19 (38.00)	23 (56.10)	185 (41.11)
* Very good*	21 (20.79)	45 (17.44)	6 (12.00)	4 (9.76)	76 (16.89)
* Excellent*	2 (1.98)	28 (10.85)	10 (20.00)	2 (4.88)	42 (9.33)

* Kruskal-Wallis test with post-hoc pairwise comparison using Dunn’s test; superscript letters indicate significantly different means

^1^ Average household size corresponds to the average number of inhabitants per household

Dietary assessments were undertaken with single 24-hour recalls conducted by trained interviewers in Master that were followed and supervised in each field. The reliability and validity of 24-hour recalls, which describe food consumed over 24 h, have extensively been used and demonstrated in several studies to assess the quality of diet [9, 29, 85–88]. A single 24-hour dietary recall was used to collect information on all food and drinks consumed by participants over the preceding day. Participants were asked to breakdown ingredients in meals. To ensure accuracy, the multi-pass method was employed [87, 89, 90]. Interviews took place at participant’s households or wherever they most felt comfortable (e.g., their office, dispensary, coffee shops, etc.).

The 24-hour recall protocol developed and stabilized by the Food and Agriculture Organization of the United Nations (FAO) in its guide on dietary diversity was conducted according to their guidelines [86]. Data were not collected on festive days since these were not considered to be representative of habitual eating practices. Self-rated health and other socio-demographic data of participants were also collected, e.g., age, occupation, marital status, main area of residence. A detailed explanation of the complete questionnaire [91] and related data collected for this study [92–95], is made freely available in Nakala, a repository dedicated to humanities and social sciences research data in France.

### Dietary diversity score (DDS)

As per FAO guidelines [86], neither the frequency of consumption nor the amount of food consumed was taken into consideration. Instead, food items were classified into one of 18 food groups to calculate the dietary diversity score (DDS).

$$\:\text{D}\text{D}\text{S}=\sum\:{\text{F}\text{G}}_{i}$$

This measure, which was calculated by summing the number of unique food groups (FG_*i*_) cited by each individual, has been extensively used and shown to be an appropriate proxy for assessing diet quality and adequacy, as well as predicting energy and nutrient intake [26–31, 86]. Participants were asked to list all ingredients included in the elaboration of mixed dishes and preparations so that they may be assigned to a specific food group. The choice of 18 food groups (Annex 1) was based on those listed by the FAO [86] with the contribution of those from the World Health Organization [96], i.e., sentinel fried foods and processed meats. Due to our focus on diet-related diseases like diabetes, obesity and hypertension, we did not distinguish vitamin A-rich food items. Further, we discriminated pulses and legumes from oleaginous fruits and nuts and alcoholic beverages from spices, condiments and hot drinks. A detailed explanation of each food group (listed in Annex 1), and their classification with examples of food items are also accessible in the Nakala data repository [91]. The dietary diversity score (DDS) was used as discrete quantitative variables and was also divided into terciles to distinguish diets of high, medium, and low diversity.

### Self-rated health (SRH)

As different studies have found SRH to be a valid proxy for individuals’ perceptions of health across cultures [25, 97], and a medium and long-term predictor of morbidity and mortality [25, 48], this measure was used for cross-cultural comparisons. Self-rated health was obtained from five possible answers to the question: “Overall, would you say that your health is: (a) excellent, (b) very good, (c) good, (d) fair or (e) poor?” Answers were subsequently dichotomized per Jylhä’s reflection showing a break between good health – “the baseline that does not normally need to have a cause” – and less than good health [25]. Hence, “excellent”, “very good”, and “good” responses were scored 0 (i.e., good health), whereas “fair” and “poor” answers were scored 1 (i.e., poor health) [46].

### Statistical analyses

Broken down by region (Table 1), demographic and socioeconomic variables are described as medians with interquartile range (IQR) when continuous or frequencies (*n*) with percentages (%) when categorical. These descriptive statistics are based on communicated responses and do not take account of answered questions. Multivariate statistical analyses were performed in the R Computational environment using the built-in “stats” package [98] and “vegan” package [99]. Univariate statistics were performed in PSPP statistical software [100]. Parametric univariate analysis of variance (ANOVA) and Student’s t-test were performed for mean comparisons where data met underlying assumptions), in which cases no data transformations were required. Normality was verified by assessing residuals for skewness and kurtosis and their Q-Q plots for normality, whereas homoscedasticity was verified using Levene’s test of homogeneity of variance. For data that did not meet underlying assumptions, nonparametric Kruskal-Wallis tests with post-hoc pairwise comparisons using Dunn’s test with the Bonferroni *p*-value adjustment method were used for mean comparisons. A one-way ANOVA with post-hoc pairwise comparisons using Tukey’s test for multiple comparisons was performed to compare mean DDS between participants’ multistate responses to SRH (i.e., poor, passable, good, very good and excellent). In contrast, a Student’s t-test was performed to compare DDS means once participant responses have been dichotomized (i.e., poor = 1 vs. good = 0). Binomial logistic regressions were used to assess the extent to which various factors, continuous and categorical (Table 2), predicted poor self-rated health: these results are presented as odds ratios (OR) with their 95% confidence intervals.

Transformation-based principal component analysis (tb-PCA), described by Legendre and Gallagher [101], was used to analyze dietary profiles and patterns. Accounting for the large presence of zero, a matrix summarizing the presence (1) or absence (0) of food groups cited in 24-hour recalls was transformed using Hellinger’s transformation before performing PCA. The inflexion point in the variation explained by each principal component was assessed in a scree plot to determine the number of axes to represent visually, to which two more principal components were added to determine factor loadings. These were obtained by correlating the original variables (i.e., food groups) with the principal components. Food groups were considered to contribute highly with a principal component if the absolute value of their Pearson’s coefficient of correlation was above 0.05.

The statistical significance of all analyses was established at α = 0.05. Nonparametric tests of significance for multivariate analyses were carried out by permutations (100,000). In contrast, parametric tests of significance for univariate analyses were carried out using the normal distribution after verifying that the data met the required assumptions.

## Results

Overall, 465 participants were interviewed individually, despite the number of participants varying considerably from one socio-ecosystem to the next (i.e., from 50 to 260). A description of demographic and socioeconomic characteristics is presented in Table 1.

The average ages of participants appeared relatively similar, but a Kruskal-Wallis test revealed that interviewed participants were older in Caribbean Coast (Guadeloupe) and Estarreja (Portugal) (*p <* 0.01). Overall, male participants were slightly under-represented, with a sex ratio (men/women) of 0.70. A Kruskal-Wallis test found statistically significant differences between average household size (*p* < 0.01), which varied widely from large (over 9 individuals) in Tessekere (Senegal) to small (2 to 3 individuals) in Caribbean Coast (Guadeloupe) and Estarreja (Portugal). Roughly half of participants lived in a conjugal union, except for participants from Tessekere (Senegal), for whom this was the case for the majority (> 90%). Despite a majority of participants declaring a salaried occupation, agriculture was an important activity in Estarreja (Portugal) and Oyapock (French Guiana), whereas livestock farming was the most important occupation in Tessekere (Senegal). In this region, around one fifth of participants declared being homemakers. In all regions, the majority of participants declared preparing meals very frequently or all the time, with an overwhelming majority in Estarreja (Portugal) and a little under half the participants in Tessekere (Senegal). The highest proportion of participants consuming a meal outside the home during the 24-hour recall period was in Caribbean Coast (Guadeloupe) (40.38%), whereas this was lowest in Tessekere (Senegal) (17.82%).

### Food groups and dietary patterns

#### Food groups

**Fig. 2:**
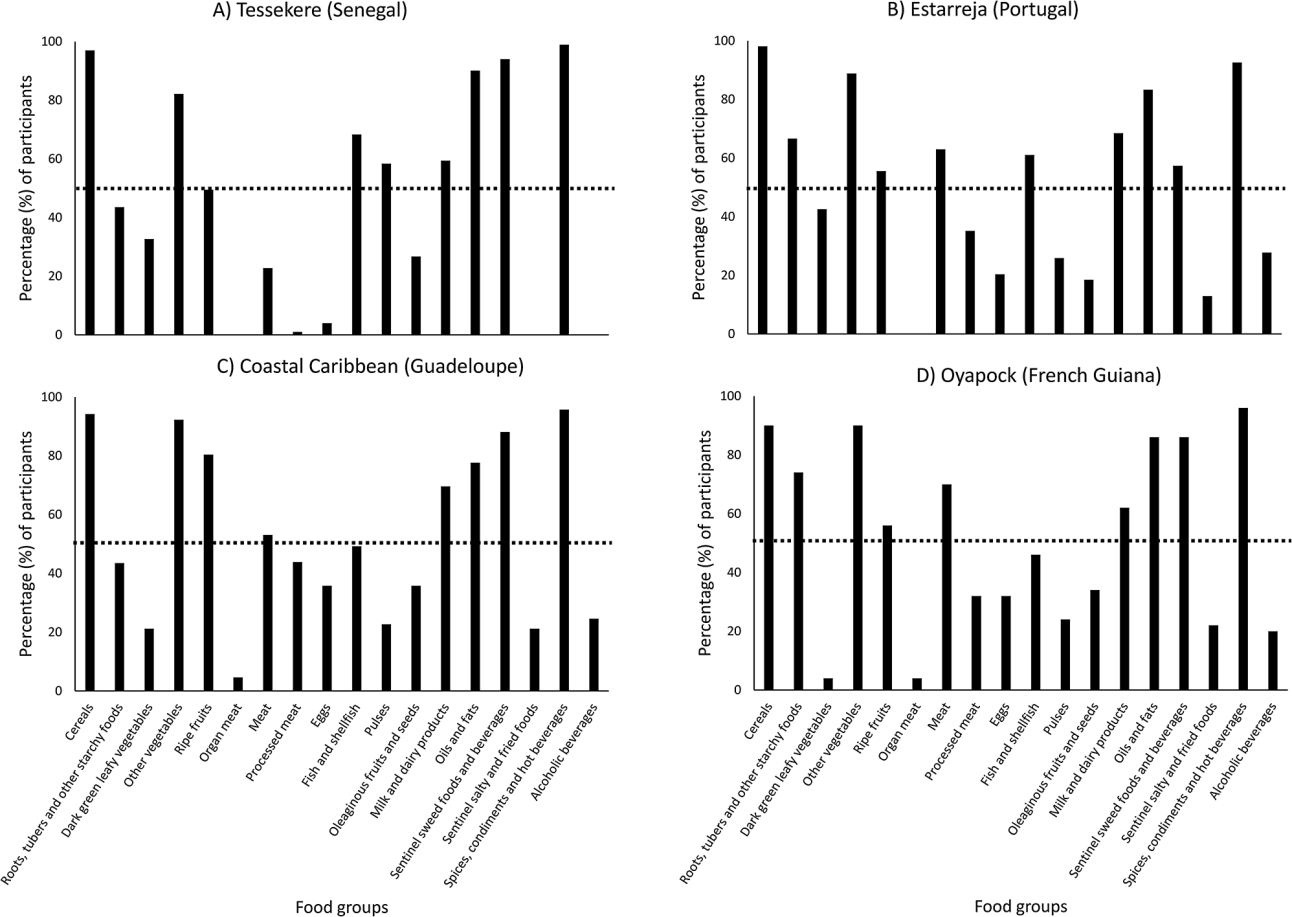
Percentage of participants consuming specific food groups declared in 24 h-dietary recalls conducted in four different socio-ecosystems. The dotted line indicates the threshold for food groups consumed by over 50% of surveys

When all regions are considered, ‘6. organ meats’ was the least cited food group, whereas ‘17. spices, condiments and hot beverages’ and ‘1. cereals’, were the most cited (Fig. 2). Some food groups appeared to differentiate some regions from others. For example, the consumption of ‘9. Eggs’, ‘7. Meat’, ‘8. processed meat’, ‘16. sentinel fried and salty foods’, ‘18. alcoholic beverage’ is much lower, if not absent, in Tessekere (Senegal) with regards to other regions. In fact, participants from Tessekere (Senegal) did not cite three food groups, namely ‘6. organ meat’, ‘16. sentinel fried and salty foods’ and ’18. alcoholic beverages’ (Fig. 2). Estarreja (Portugal) was the only other region where specific food groups were not cited by any of the participants interviewed, namely ‘6. organ meat’. The consumption of ‘3. dark green leafy vegetables’ was lowest in Oyapock (French Guiana). On the other hand, ‘11. pulses’ were characteristic of Tessekere (Senegal), the region with the highest citation, whereas participants from Caribbean Coast (Guadeloupe) cited ‘5. ripe fruits’ the most. Finally, there were relatively more citations of ‘10. fish and shellfish’ consumption in Tessekere (Senegal), which is located in the sub-Saharan desert, than all other regions, namely Estarreja (Portugal) and Caribbean Coast (Guadeloupe), an island known for its diversity of seafood and vibrant commercial and recreational fisheries [102–105].

**Fig. 3:**
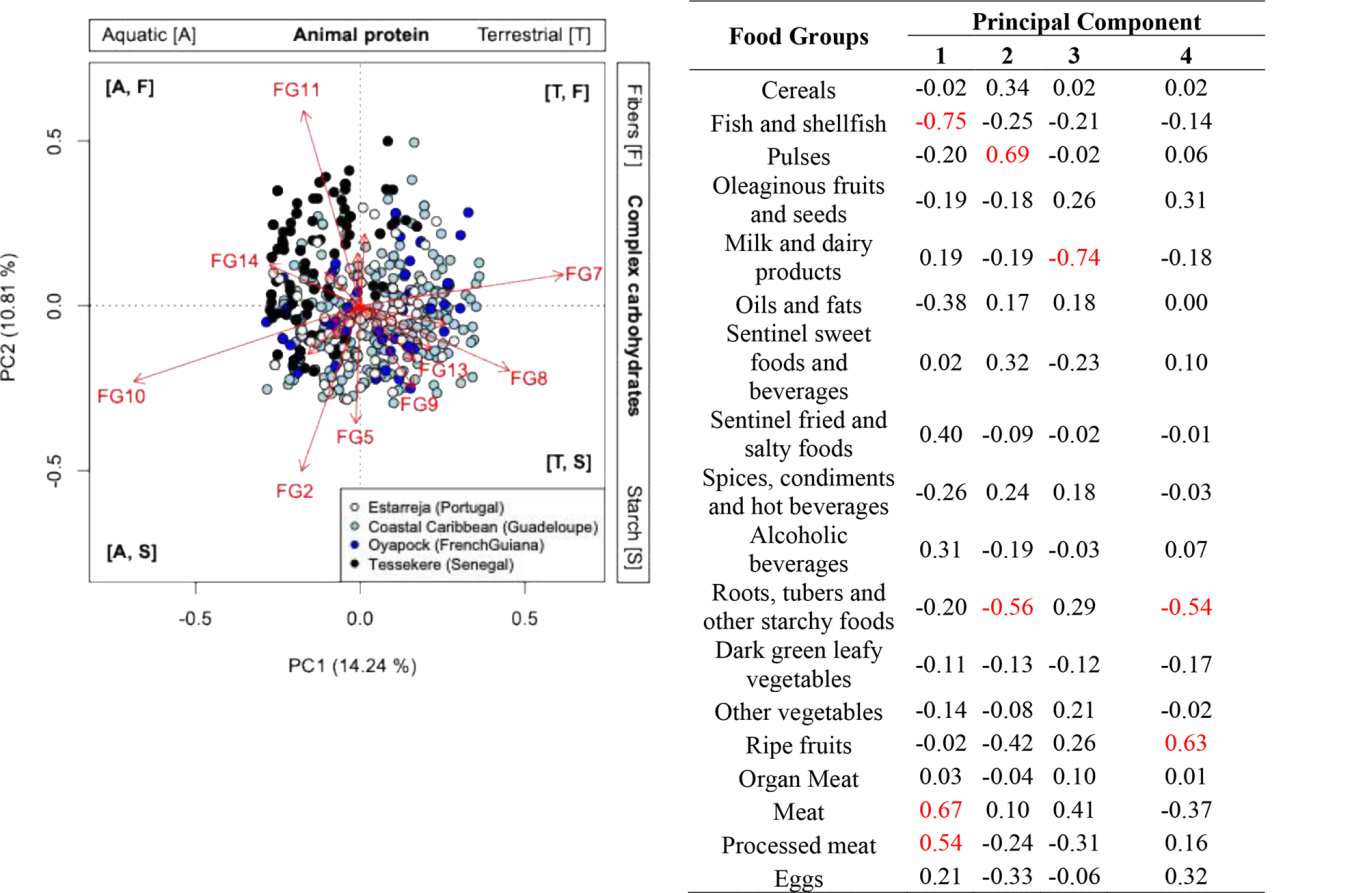
Principal component analysis (PCA) in type 2 scaling of participants consuming specific food groups declared in 24 h-dietary recalls conducted in four different socio-ecosystems (OHMi-Téssékéré, OHMi-Estarreja, OHM-Littoral-Caraïbe, OHM-Oyapock). (**A**) Visual representations of results in ordination biplots in type 2 scaling. Arrows represent food groups and symbols represent participants. (**B**) Pearson’s correlation coefficients (*ρ*) between the first four principal components and original variables. Coefficients in red were considered to be highly associated with a principal component

A PCA of participants’ dietary profiles based on cited food groups managed to explain 25.05% of the variation on its two first principal components (λ_1_ = 0.05, λ_2_ = 0.04) (Fig. 3). Food groups that were best correlated (|*ρ*| > 0.50) with the first principal component (PC1) include ‘10. fish and shellfish’ in opposition to ‘8. processed meat’ and ‘7. meat’, therefore reflecting a gradient representing the main sources of animal protein from aquatic to terrestrial origin. The second principal component (PC2) correlated best with ‘11. pulses’ and ‘2. roots, tubers and other starchy foods’, therefore reflecting a gradient representing predominant sources of complex carbohydrates from high fiber to high starch food items. Furthermore, participants from Tessekere (Senegal) appear to cluster together based on an increased citation of food items in groups ‘10. fish and shellfish’, ‘14. oils and fats’, ‘11. pulses’ and ‘15. sentinel sweet foods and beverages’. Using the indicative gradients of the first two principal components for naming, this food pattern was labeled “animal protein of aquatic origin (i.e., fish and shellfish) and high fiber”.

Participants from Caribbean Coast (Guadeloupe), Oyapock (French Guiana) and Estarreja (Portugal) appear to overlap considerably more, but with participants from Caribbean Coast (Guadeloupe) and Oyapock (French Guiana) slightly more offset from the center than participants from Estarreja (Portugal), thus appearing opposite to participants from Tessekere (Senegal). Food items that appear to characterize participants from Caribbean Coast (Guadeloupe) and Oyapock (French Guiana) belong to the food groups ‘7. meat’, ‘8. processed meat’, ‘9. eggs’, ‘5. ripe fruits’ and ‘2. roots, tubers and other starchy foods’. These food patterns are labeled “animal protein of terrestrial origin (i.e., mammals and birds) and high starch”. Because participants from Estarreja appear in the center of these opposing food typologies, their food pattern was labeled “mixed animal proteins and complex carbohydrates”.

#### Dietary diversity scores (DDS)

**Fig. 4:**
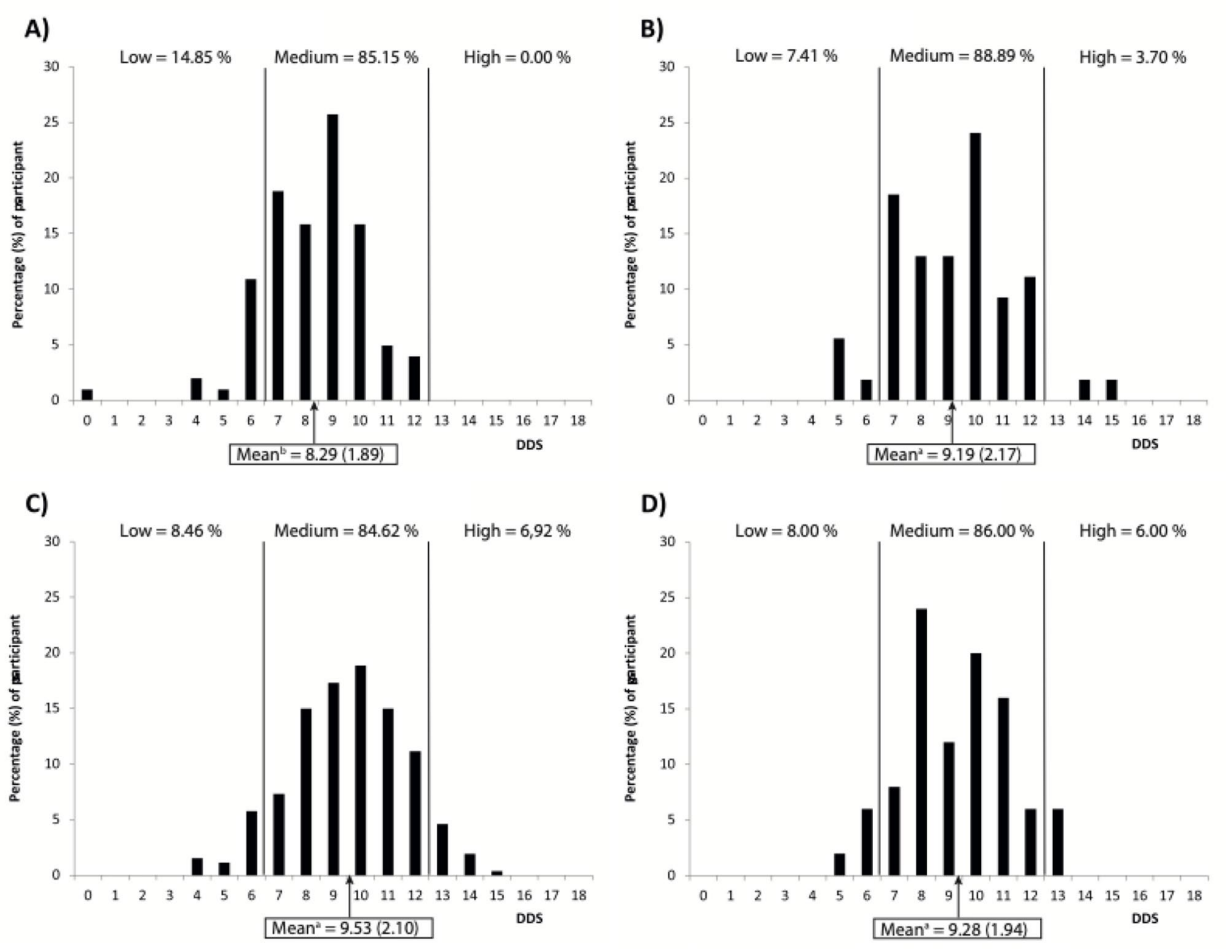
Mean (standard deviation) dietary diversity score (DDS) and DDS distribution among participants from (**A**) Téssékéré (Senegal), (**B**) Estarreja (Portugal), (**C**) Coastal Caribbean (Guadeloupe) and (**D**) Oyapock (French Guiana). Distributions of DDS are marked by tercile cut-offs. Differences between means were statistically significant (*p* < 0.01) in a one-way ANOVA. Letters indicate statistically different means based on *post-hoc* pairwise comparisons

Overall, the mean DDS was 9.20, reflecting a medium score. Averages for all regions remained in the second tercile (i.e., medium score) of dietary diversity, yet an ANOVA found differences between means to be statistically significant (*p* < 0.01; Fig. 4). Multiple comparisons reveal that this is due to Tessekere (Senegal) possessing the lowest mean DDS whereas those for the other regions were not statistically different from one another (Fig. 4). One person from Tessekere (Senegal) declared not having eaten for 24 h. Although this participant might be considered an outlier, it was not removed from the analysis. While Tessekere (Senegal) was the only region where none of the dietary diversity of participants scored in the highest tercile, it represents the region with the highest proportion of participants scoring in the lowest tercile (Fig. 4a). On the other hand, proportionally more participants from Estarreja (Portugal) and Caribbean Coast (Guadeloupe) scored in the medium tercile and the highest tercile, respectively (Figs. 3c and 4b). On average, DDS was highest in Caribbean Coast (Guadeloupe), followed by Oyapock (French Guiana), Estarreja (Portugal) and finally Tessekere (Senegal) (Fig. 4).

### Self-rated health and diet

**Fig. 5:**
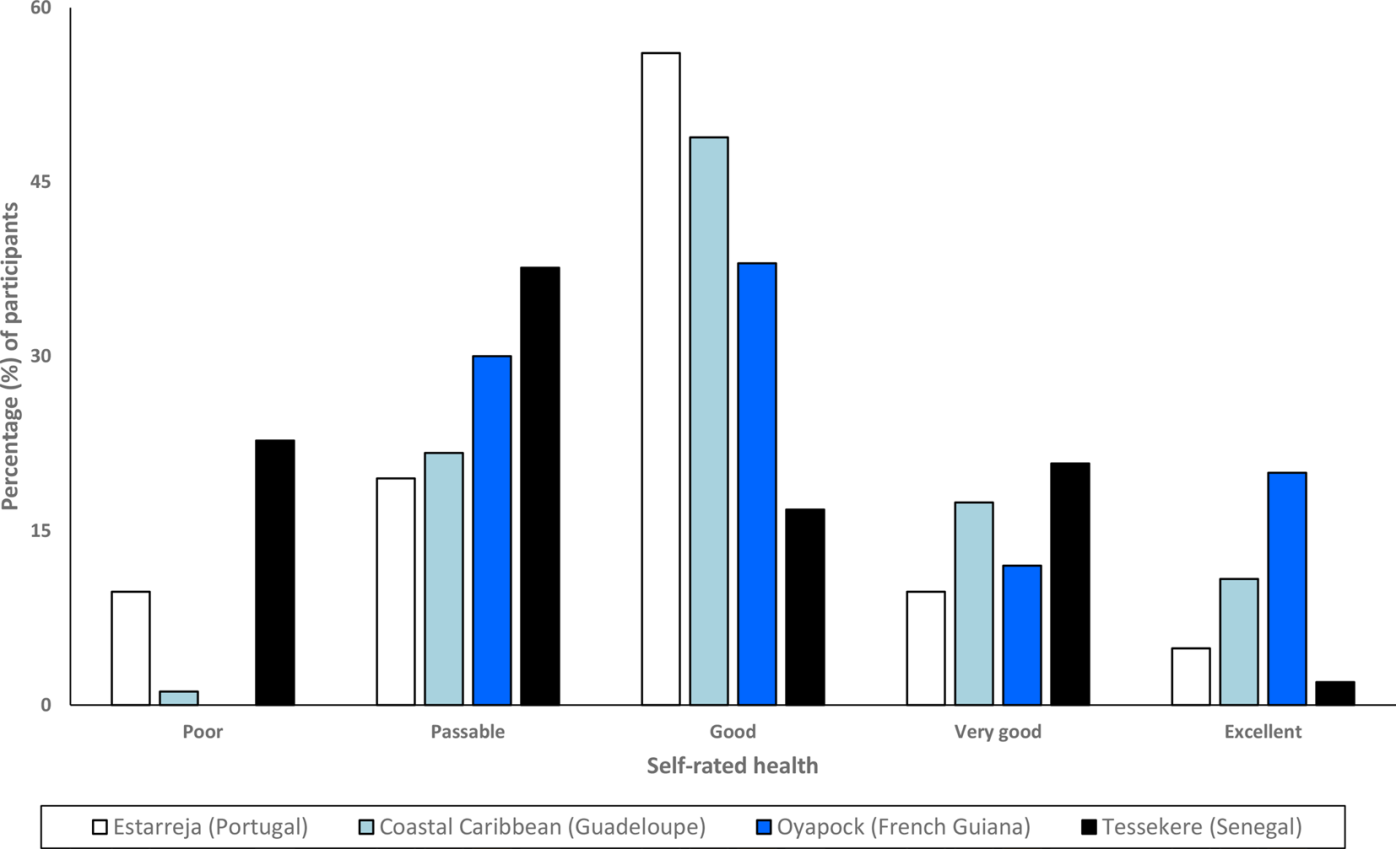
Self-rated health by OHM(i)

More than one-third of participants reported their health as ‘good’ (41.11%), whereas ‘bad’ and ‘excellent’ health were the least reported, at 6.67% and 9.33%, respectively (Table 1; Fig. 5). Estarreja (Portugal) had the most participants reporting “good” health, and Oyapock (French Guiana) had the most participants reporting “excellent” health (Table 1; Fig. 5). Participants from Tessekere (Senegal) rated their health more poorly than all other regions and was the only one where more than half of participants rated their health as “poor” or “passable” (60.39%) (Fig. 5). Inversely, Caribbean Coast (Guadeloupe) had the highest percentage of participants reporting “good”, “very good”, or “excellent” health (77.13%) (Table 1; Fig. 5).

**Fig. 6:**
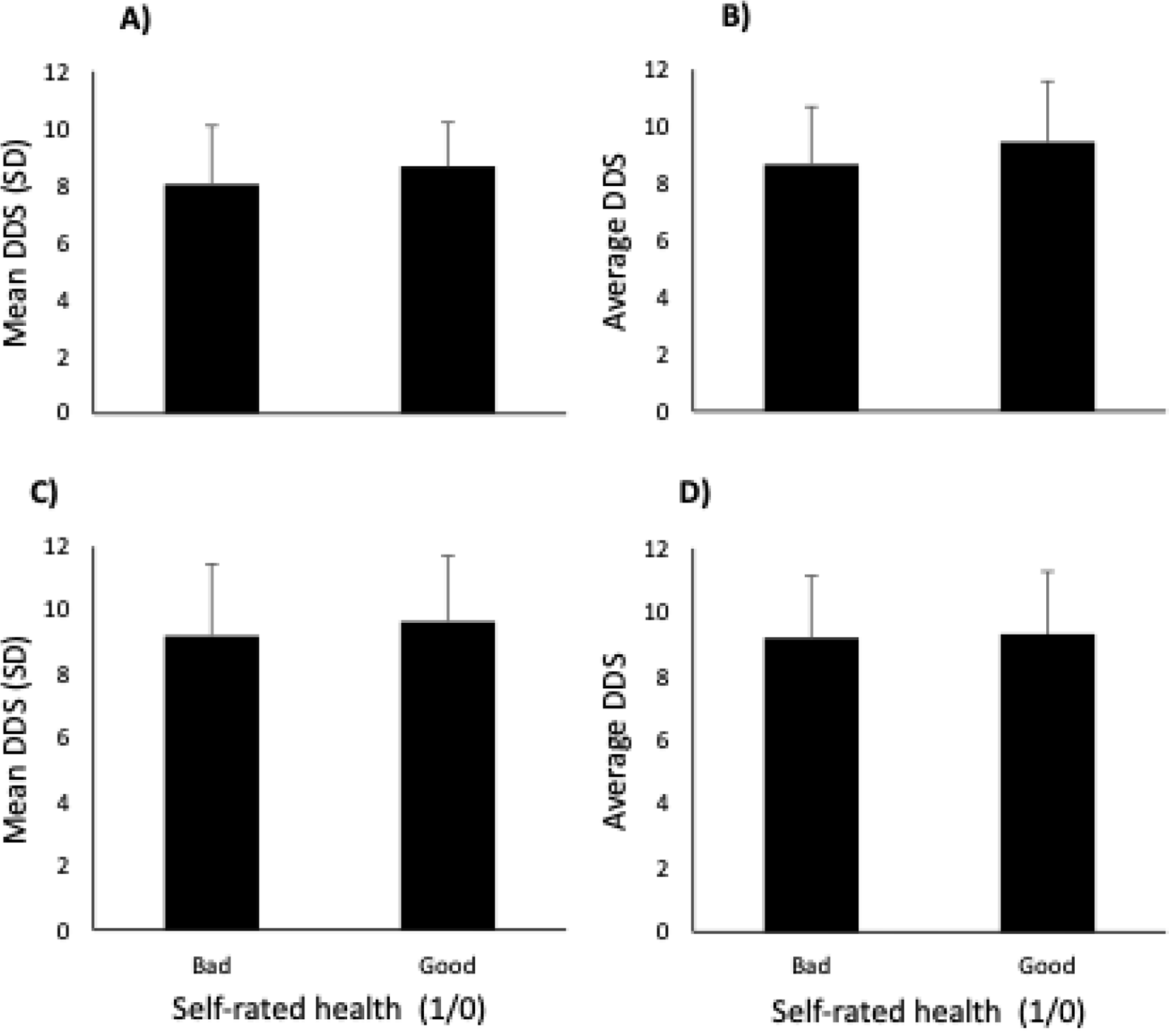
Dietary diversity score (DDS) and dichotomized answers to self-rated health (SRH) from four different socio-ecosystems: (**A**) Téssékéré (Senegal), (**B**) Estarreja (Portugal), (**C**) Coastal Caribbean (Guadeloupe) and (**D**) Oyapock (French Guiana). Bad (1) includes “poor” and “passable”, whereas good (0) includes “good”, “very good”, and “excellent”. Bars represent the mean DDS and standard deviation (SD) for each SRH response category. Mean DDS between bad (1) and good (0) were statistically significant in a one-tailed t-test (*p* < 0.01). A two-way ANOVA indicated no interactions between SRH and the four socio-ecosystems (*p* = 0.88)

Overall, there was an incremental increase in mean DDS with increased self-rated health (SRH) levels (data not shown); a one-way ANOVA found this relationship to be statistically significant (*p* < 0.01). Multiple comparisons indicate that DDS was significantly higher among participants declaring “good”, “very good” and “excellent” health. This relationship is further supported by a one-tailed t-test that found that DDS was lower among participants declaring “bad” health (*p* < 0.01) once responses were been dichotomized (Fig. 6). A two-way ANOVA revealed that there were no statistically significant interactions between the participants’ regions of residence and SRH, whether SRH was dichotomized (*p =* 0.88) or not (*p* = 0.19). Despite differences in mean DDS between regions (Fig. 4), this does not affect relationships between DDS and SRH from one region to another (Fig. 6).

This is further supported by a binary logistic regression that found a statistically significant relationship in DDS predicting SRH (*p* < 0.01; Table 2). When considered in a more complex model containing socio-demographic variables (Table 2), DDS was found to be statistically significant in predicting SRH along with age and one’s level of participation in preparing meals (Table 2). The odds ratio of SRH with sex (0.61) reveals that women are more likely than men to declare bad health. However, this relationship was not considered statistically significant as the p-value was just above the rejection threshold (*p =* 0.053). Relationships with statistically significant factors are interpreted as follows:

- as DDS increases, the probability of participants declaring bad health decreases;
- as age increases, the probability of participants declaring bad health increases;
- participants are incrementally more likely to declare bad health when preparing meals sometimes and never rather than those who always cook.
- Participants are incrementally less likely to declare bad health when residing in Oyapock (French Guiana), Estarreja (Portugal), Caribbean Coast (Guadeloupe) as opposed to Tessekere (Senegal).
- Regardless of the region considered, the participants’ age, sex, or inclination to prepare meals at home, the higher the dietary diversity score of individuals, the higher their chances of declaring themselves in good health.

**Table 2:** Adjusted odds ratios (OR) for poor self-rated health all OHM

Variables	Categories	*p*-value	Odds ratio	CI for OR (95%)	
				Minimum	Maximum
*Model 1*					
DDS	Continuous	< 0.01**	0.83	0.75	0.92
*Model 2*					
DDS	Continuous	0.03*	0.88	0.79	0.99
Age	Continuous	< 0.01**	1.04	1.02	1.05
Household size	Continuous	0.68	0.99	0.93	1.05
Sex	Men Reference: Women	0.05	0.61	0.37	1.01
Marital Status	Conjugal union	0.73	1.21	0.40	3.65
	Single Reference: widowed	0.50	1.47	0.48	4.58
Meal preparation	Sometimes	0.07	1.66	0.96	2.87
	Never Reference: All the time	< 0.01**	3.31	1.55	7.07
Out of home meal	No Reference: Yes	0.62	0.88	0.53	1.46
OHM	Estarreja	0.02*	0.30	0.11	0.80
	Littoral Caraïbe	< 0.01**	0.15	0.07	0.33
	Oyapock Reference: Tessekere	0.01*	0.33	0.14	0.80

* *p* < 0.05

** *p* < 0.005

## Discussion

### Cultural food patterns between the four ecosystems studied

Using the method of Auma et al. [87]. for characterizing food patterns, our analysis reveals a pattern distinguishing the source of animal protein opposing fish/seafood-rich diets from meat-based diets along PC1 (Fig. 3). This gradient seems to distinguish Tessekere (Senegal) from Caribbean Coast (Guadeloupe) and Oyapock (French Guiana), respectively. PC2 reveals a food pattern distinguishing the source of plant-based carbohydrates opposing pulses from starchy tubers and other non-cereal starchy foods in a gradient similar to PC1. Unlike the analysis of Auma et al. [87], these patterns do not capture dietary transitions at first glance. Still, they keep with some of the cultural characteristics of each socio-ecosystem’s food system.

Pastoralism is an important practice in Senegal, with important implications for an individual and a family’s economic and social capital [106]. Despite the large herds of sheep, goats and cattle characteristics of the Sahelian landscape, their contribution to food security comes less from their consumption than from their wealth and status [107]. Indeed, fish is the centerpiece of Senegal’s national dish, *thieboudien* (i.e., fish and rice) which has been inscribed on UNESCO’s Representative List of the Intangible Cultural Heritage of Humanity [108]. Fish and seafood reach the local markets of the Sahel where it is purchased fresh, dried or smoked. Not only are the latter two more suitable for better preservation in the sub-Saharan desert, but their organoleptic properties provide the unique taste of regional dishes. Despite Portugal being the second biggest consumer of fish and seafood in Europe [109–111], with Estarreja’s (Portugal) proximity to the coast ascribing it to a strong fishing heritage, the importance of fish in the dietary recalls came only second to Téssékéré, thus highlighting the importance of this food group to sub-Saharan dietary practices.

In Oyapock (French Guiana), as in Caribbean Coast (Guadeloupe), fish consumption is low, in contrast to the consumption of meat products. This is to be expected from Oyapock (French Guiana), whose culturally diverse populations have an important historical tradition of hunting [112]. For Caribbean Coast (Guadeloupe), on the other hand, this likely acts as an indicator of dietary transitions as the insular nature of this Caribbean Island has historically had a significant impact on shaping the food system with a heavy reliance on fish and seafood. The importance of processed meats gives credence to dietary transitions, and the superposition of participants from Oyapock (French Guiana) with those from Caribbean Coast (Guadeloupe) suggests that the former may be experiencing a similar transition to the latter.

The gradient on PC2 also reveals socio-ecosystem characteristics. Pulses are an essential element of Senegalese cuisine, particularly niébé, whereas non-cereal starchy foods, namely tubers, are an essential element of the Caribbean and Amazonian food systems. This is particularly true in Oyapock (French Guiana). Consistent with other regions of the Amazon [9, 113–118], cassava consumption is high in French Guiana, especially in the Oyapock region, where a roasted cassava semolina, known as *kwak* in Creole and *farinha* in Brazilian Portuguese, is a staple of Amazonian Indigenous, Creole and Brazilian cuisine [18]. However, this is not the case in Caribbean Coast (Guadeloupe), where more than half of the population consumes neither pulses nor non-cereal starches.

### Self-rated health and dietary diversity score

Overall, results indicate that, regardless of age, sex, marital status, household size, meals eaten outside the home, the frequency of preparing meals, and especially the regions considered (and consequently, their socio-cultural characteristics), self-rated health is significantly associated with dietary diversity. To the best of our knowledge, this study is the first to demonstrate this by assessing the relationship between DDS and SRH. Credence for these results is provided by multiple studies that support the relationships obtained between SRH and different socio-demographic variables. Both cross-sectional and longitudinal analyses have found that global SRH declines with age [119, 120]. In longitudinal studies, women report worse SRH than men throughout most of adulthood [121, 122], although this disparity disappears when differences in socio-economic and health covariates are considered [122]. Results from this study support geographical disparities observed when comparing SRH between European countries [57], the United States [58], and the Senegalese sub-Sahara [46]. Furthermore, the relationship between meal preparation and SRH is supported by studies that have also found that positive eating practices, like the number of homemade meal preparation episodes or the time spent preparing meals at home, are linked to better SRH [80], and self-rated mental health and stress [78, 79], respectively. Given that DDS has also been shown to capture socio-economic status and household food security [123, 124], it may also capture many other determinants of health that impact SRH. For example, Poorrezaeian et al. found that DDS was inversely associated with depression [31] and anxiety [125]. In turn, Cabiedes-Miragaya et al. [126] identified several lifestyle habits, such as the consumption of sweets and fruits and social interaction around meals – which greatly influence eating behaviour [127] – as being associated with subjective well-being (i.e., perceived health, life satisfaction, and feeling of happiness).

Due to health implications implied by SRH, this infers that DDS is linked to medium- and long-term morbidity and mortality in the regions implicated in this study. The declaration of poor self-rated health by a patient could thus prompt healthcare professionals to consider associated modifiable risk factors such as diet. As diet is intrinsically linked to health, it therefore seems beneficial, in the case of poor self-assessments of health, to promote the diversification of food types in meals, to recommend dietary interventions with nutritionists, and to identify potential nutritional imbalance. In addition, the significant association between DDS and SRH offers practical value for interpreting dietary diversity calculated as an indiscriminate cumulative measure of food groups. For individuals living with diet-related diseases, such as diabetes, unhealthy and ultra-processed foods may already constitute a substantial part of their dietary profile [128]. While reducing their consumption is a crucial objective of nutrition therapy in managing diabetes, increasing dietary diversity is equally important to improve overall nutrient intake and health outcomes [16]. In this context, SRH can serve as a valuable subjective metric for healthcare professionals, including nutritionists and dietitians, to evaluate the adherence to, and success of, dietary interventions. SRH may reflect not only the individual’s perception of their health improvements but also their satisfaction and motivation regarding the dietary changes, which are critical factors in achieving long-term dietary compliance.

### Nutritional and epidemiological transitions

By comparing the dietary diversity score and self-assessed health in four different socio-ecosystems, this study provides a clearer picture of the dietary and epidemiological transition processes within these systems. As in many populations at the start of the nutritional transition [7, 10, 129], 24 h recalls suggest that meat is consumed more rarely in the Senegalese Ferlo, whereas it is consumed by more than half the individuals in the other socio-ecosystems (Fig. 2). Yet in this remote region of Senegal, 24 h recalls also suggest that. legumes are consumed by a greater number of people, thus complementing protein and fiber intake. Nonetheless, the population of Tessekere in Senegal’s Ferlo region had a significantly lower dietary diversity score than the others regions (Fig. 4). Public and private (NGO) initiatives linked to the Great Green Wall have aimed at improving local food diversity, notably through the establishment of integrated community agricultural farms. These initiatives continue to be challenged by issues related to infrastructure and access to water, and local nutrition relies for the moment on the consumption of a few food groups. Despite previous studies that have documented changing attitudes around the consumption of sentinel fried foods like chips (or crisps) and the observation of certain eating norms [130], the consumption of sentinel fried food and processed meats, in general, were not frequently cited in comparison to the three other regions. Considering the low prevalence of diabetes and obesity [131] in the Ferlo, results do not suggest that this region has transitioned into a dietary pattern favourable for the development of chronic diseases. In keeping with the region’s famine of the 1970s and the ongoing desertification of the Sahel [106], the Ferlo People may still be on the cusp of transitioning out of a dietary pattern that Popkin describes as “receding famine” [7]. This may also explain why participants from Tessekere (Senegal) were more likely to declare bad health than the other three regions.

In Caribbean Coast (Guadeloupe) and Oyapock (French Guiana), DDS are similar and highest with meat consumption as an important indicator of their dietary typology. For people living along the Oyapock River, particularly Indigenous Peoples, hunting is a strong cultural marker, with game considered food *par excellence* [112]. Farm-raised animals like beef, pork and chicken are important markers of Brazilian and Creole dishes, such as *fricassé* and *colombo* [132], types of stews also feature in Creole cuisine from Guadeloupe [133, 134]. As opposed to northern Portugal and Senegal’s Ferlo region [135, 136], the prevalence of diabetes and obesity is higher in French Guiana and Guadeloupe, and among the highest in all of France [137–139]. This provides further evidence of the nutrition and food transition that have been documented in these regions [18, 132, 140–142]. The higher likelihood of declaring good health may be due to the insidious nature of chronic diseases like diabetes and obesity. The negative health impacts of these illnesses are felt with time, progressing in severity throughout an individual’s lifetime [143]. This relationship may very well be captured by results indicating that the likelihood of declaring bad health increases with age (Table 2).

Finally, food patterns in Portugal, despite being located on the North Atlantic Ocean, are generally ascribed more specifically to the Mediterranean dietary pattern where it is not only considered a historical and cultural asset, but also a legacy to preserve [144–146]. This dietary pattern is considered diverse with fish and leafy greens being relatively important in the consumption of animal and plant foods [146, 147], which this study corroborates (Fig. 2). While this also implies that DDS should be significant in Estarreja (Portugal), it was comparable to those for Caribbean Coast (Guadeloupe) and Oyapock (French Guiana) in this study (Fig. 4). However, the latter also accounts for sweet and salty sentinel foods, which appear to be consumed much less in Estarreja (Portugal), and therefore contribute less to DDS (Fig. 2). Considering the health benefits associated with the Mediterranean diet and its promotion in Portugal [144, 146], this is consistent with the 5th pattern (i.e., behavioral changes) of Popkin’s nutritional transition model [7], as previously suggested [62].

Finally, several results obtained in this study put the link between dietary diversity score and self-rated health into perspective. While DDS is frequently used in contextualized studies of problems related to undernourishment, it is much less frequently used in “classic” or over-nutrition contexts. In a “classic” context involving four different socio-ecosystems, the calculation of a DDS allowing a comparison of these different regions was able to highlight a major result: regardless of the locality considered, age, sex, marital status, meal preparation method and number of meals eaten out, the dietary diversity score is significantly linked to self-rated health. In line with the study published in Australia by Collins et al. [148], better SRH is linked to a higher DDS. Thus, SRH, which is lower among women, the elderly [55, 149], people who don’t prepare their meals at home and in Tessekere (Senegal), is also lower among people with lower dietary diversity.

If a parallel were to be drawn between dietary diversity score, health and nutritional transition, it would be reasonable to think that the Senegalese territory studied presents the characteristics of the first stages of this transition: low diversity, rare diseases linked to overnutrition and poor health. In Guadeloupe and French Guiana, indigenous and introduced foods coexist, interact and may hybridize, as other studies have shown [62, 140]. Ultra-processed foods are common, and their impact on health, particularly the high prevalence of chronic diseases, aligns with Popkin’s nutrition transition model. In contrast, Estarreja (Portugal)’s current food system manages globalization relatively well. But to suggest that it has reached the final pattern of the nutrition transition (i.e., behavioral changes) does not protect it from evolving from the potential negative effects of food globalization on health.

However, it is important to note, as De Lima et al. [9] argue, “that the nutrition transition model should not be assumed as a generalized and homogeneous global process”. Due to multiple factors involved in the process of the nutrition transition – e.g., social, cultural, historical, economic and environmental – changes in diet are not fully understood [9]. Dufour and Bender [150] discuss limitation to the nutrition transition model, and studies must therefore be attentive to the specific features of the territories they study, paying attention to the comparability of territories, as in this work, and to the specific characteristics of each socio-ecosystem.

### Limitations

There are several limitations to this study, which include most notably the varying sample sizes between socio-ecosystems. This reflects difficulties in conducting research that is inherently unique to each highly contrasted socio-ecosystem.

There are several well-known methodological limitations to the use of 24-hour dietary recalls. This method is declarative relying on people’s memory with the risk of misreporting. Any interpretations of DDS calculated here are subject to the same limitations that apply to 24-hour recalls. The advantage of the 24-hour recall survey is that it is simple, quick and inexpensive [151, 152], it allows for the calculation of dietary scores, which are rapid and efficient means to estimate nutrient adequacy [28, 29, 151, 152], and to assess changes in diet through time [28, 33]. This makes it more easily interoperable, and the value of the data produced here will undoubtedly increase when reproduced periodically over the long term.

Finally, there are no conventionally defined numbers of food groups to consider when calculating DDS [86]. This may be interpreted as a limit to the use of DDS or inversely seen as an advantage as varying definitions of food groups from one study to the next reflect particularities of each food systems. Constraining these to standardized food groups for cross-sectional comparisons is an exercise in balancing the conservation of information. Many studies calculate DDS using nine to 12 food groups [153], whereas the FAO and WHO guidelines, on which this study’s list is based, describe 16 and 18 groups, respectively [86, 96]. To facilitate the cross-cultural comparison of dietary diversity between socio-ecosystems with distinctive food systems, a relative DDS scaled according to each system’s maximum score may be considered.

## Conclusion

This study is, to the best of our knowledge, the first to show a positive association between DDS and SRH across distinct cultural groups, using qualitative data collection methods and quantitative analyses. If only a few elements were to be highlighted, they would be that: (i) diverse diets containing a variety of different foods are linked to increased subjective health; (ii) this remains true despite differences in dietary diversity between socio-ecosystems; and (iii) this association indiscriminately accounts for sentinel unhealthy foods, which underscores the utility of SRH as a tool for quickly and qualitatively assessing nutrition therapy outcomes.

Moving forward, the questionnaire used in this study can be employed repeatedly and throughout different periods of the year to obtain a more representative picture and holistic view of food systems and typologies [33, 151, 152]. This could be complemented by other types of nutritional surveys like the food frequency questionnaire [154, 155]. Simply repeating this study at regular intervals can yield crucial insights into nutrition and food transitions, and the evolution of food systems over time. While the simplicity of DDS offers practical advantages, careful consideration should be given when using it to study its relationship with SRH in greater depth. Simple transformations may be applied to DDS to account for regions with diverse and distinctive foodscapes (i.e., relative-DDS) and the nutritional weight given to unhealthy foods (i.e., directional-DDS). This could enhance our understanding of how food systems move in and out of patterns linked to overnutrition-related chronic diseases. Despite these considerations, the findings here further further strengthen the utility of dietary scores. The positive relationship between DDS and SRH highlights that even food consumption at a given moment in time has an equally important role in the subjective perception of health as dietary habits over the long term.

### Annex

**Annex 1:** Categories for the classification of food items, levels of processing and the sourcing of food items

Category	FAO^a^	WHO^b^
**Food group**		
1. Cereals	X	X
2. Roots, tubers and other starchy foods	X	X
3. Dark green leafy vegetables	X	X
4. Other vegetables	X	X
5. Ripe fruits	X	X
6. Organ Meat	X	X
7. Meat	X	X
8. Processed meat		X
9. Eggs	X	X
10. Fish and shellfish	X	X
11. Pulses*	X	X
12. Oleaginous fruits and seeds*	X	X
13. Milk and dairy products*	X	X
14. Oils and fats	X	
15. Sentinel sweet foods and beverages	X	X
16. Sentinel fried and salty foods		X
17. Spices, condiments and hot beverages*	X	
18. Alcoholic beverages*	X	

a Kennedy et al. [1]

b WHO [2]

c Monteiro et al. [3]

## Electronic supplementary material

Below is the link to the electronic supplementary material.

Supplementary Material 1

## Data Availability

The data that support the findings of this study are available from CNRS, but restrictions apply to the availability of these data, and so are not publicly available. The data are, however, available from the authors upon reasonable request and with the permission of CNRS.
