# Supplementary material for: Associations between dietary diversity and self-rated health in a transverse study of four local food systems (French Guiana, Guadeloupe, Portugal and Senegal)
Source: BMC Public Health. 2025 Feb 28;25:823. doi: 10.1186/s12889-025-21872-8 (PMC11871774; doi:10.1186/s12889-025-21872-8)
Supplement: Supplementary file 1 — Supplementary Material 1 [file 12889_2025_21872_MOESM1_ESM.pdf]

User's guide for the SASI questionnaire

Etic component : Dietary Diversity Questionnaire (DDQ) - 24-hour recall

The method for collecting information on dietary diversity consists of a qualitative recall over 24 hours of all foods and drinks consumed by an interviewee. This person is asked to indicate all the foods and drinks they consumed the day before, including day and night, at home or outside. **It is important to become familiar with local names for foods, dishes, and meals and to use them when communicating with participants.**

Let the person describe the foods they consumed during the last 24 hours[1] themselves. This method is referred to as a "recall." The following steps can be taken:

- Ask the interviewee to mention all the foods and drinks (meals and snacks) consumed the day before, during the day and night (**Q1**). Begin with the first food or drink ingested in the morning and let the person list until they have finished the enumeration. Enter this information in the spaces provided in **grid 1**.

- As the interviewee indicates the foods and drinks consumed, underline them in **grid 2**, each of which represents a food group, and enter "1" in the column corresponding to each group that has at least one of its elements underlined. If a food is not listed in any group or is difficult to assign to a group, note it in the margin and consult the project's supervisor and/or resource person.

- Once the interviewee has finished listing the foods and dishes consumed the day before, review the various items mentioned. If a cooked dish is mentioned, ask for all the ingredients and enter them in both grids (**Q1a**).

- Ask if snacks were taken between main meals and if the person did not skip any meals (**Q1b**).

- Ask if any elements were added (**Q1c**), such as sugar (in tea or coffee, for example) or oil (cooked dishes or fried foods, for example).

- Once the recall is complete, list the food groups in which no elements were underlined in case they were forgotten (**Q2**). It is not necessary to provide the exact name of the food group. For example, if fruits, vegetables, or tubers were not mentioned during the recall, ask if they were consumed. Enter "0" in the right-hand column of **grid 2** when it is confirmed that no food from that group was consumed.

- Ask if any special foods were consumed (**Q3**).

- Review the foods mentioned. For each one, ask about the source of supply (**Q4**) and the degree of processing (**Q5**). Next to each food item noted in **grid 1**, enter the code corresponding to each response in parentheses. <u>Use the choices in the frame of **grid 1** to guide the participant.</u>

---

[1] For an example of this method, see Savy *et al.* (2006).

- Ask which meal was the most important. Review the foods mentioned in this meal to determine, according to the participant, if these foods are perceived as being local (or not), traditional (or not), and healthy (or not) (**Q6**). Ask these three questions in tandem to review the foods mentioned only once. Next to each food item noted in **grid 1**, enter the code corresponding to each response in parentheses. Use the choices in the frame of **grid 1** to guide the participant.

The recall method has several advantages over the method of questioning the participant by reading all the foods from all the groups of the questionnaire and asking the person to indicate for each food whether it was consumed or not:

1) It is faster.
2) It is less tedious for the interviewee, who does not need to answer yes or no for each food.
3) It allows the interviewee to actively participate in the interview.
4) It makes it easier to take into account the ingredients used in the cooked dishes.

Emic component: Food system representations

The method of collecting information on the representations of the food system consists of an interview guide containing semi-structured questions with open-ended answers. Because the purpose is to document emic representations, *i.e.*, from the participant's point of view, it is important **not** to suggest potential answers to the questions in order to evoke a response.

If possible, proceed in the order of the theme questions, as they appear in the questionnaire:

Q7+8. These questions serve to capture emic representations of good (or bad) nutrition and diet.

a. It is possible that some responses may evoke the question posed in a different formulation, *e.g.*, "Eating well means eating good quality products." Therefore, the participant should be asked to define or elaborate.

b. If specific foods or meals are mentioned, note.

Q9+10. These questions serve to capture the influencing factors (positive and/or negative) on nutrition and diet from an emic perspective.

Q11. This question serves to capture changes in diet, if any, observed by the interviewee in their lifetime. It is possible that the interviewee may share second-hand accounts, such as, "My grandparents, or my parents, said that before, there weren't all the products in the grocery store." This information is valuable and enriching, but it is important to distinguish it from observations directly from the interviewee's experience. Use follow-up questions, such as "Why?" and "How?", to encourage the development of "Yes/No" responses (**Q11a**).

Q12. This question serves to highlight the disrupting event of the OHM if it was not addressed in **Q11**. Use follow-up questions, such as "Why?" and "How?", to encourage the development of "Yes/No" responses (**Q11a**).

Q13. The interviewee may allude to diet-related health problems in answering other questions in the interview guide. This question therefore serves to identify whether this person is closely and personally concerned with these health problems.

Q14. This question serves to inform the part of the SASI project that deals with the detection and quantification of contaminants in food, *i.e.*, identifying foods to be analyzed and contaminants to be detected, and the negative links made between food and health. Due to the open-ended nature of the question, however, participants can respond with a range of answers that are not related to contamination.

Q15. This question serves to further elucidate participants' preferences for where food is sourced. Naturally, this question can lead to further elaboration on "eating well" and influencing factors based on responses.

Q16. The final word! This question gives the respondent the chance to share information, opinions or any other details that the respondent feels are relevant.

Some important notes on the emic component:

- Although there is a sense in the order of the questions, a participant may not answer a question directly, but rather address another topic in the questionnaire. This does not matter. It is important to adapt to the participant's response and eventually guide him or her back to the original question.

- Take your time and let participants respond and think. Some people choose their words well and think long and hard before answering. A pause on the part of the participant does not necessarily imply that the answer is complete. Count a few "Mississippi" in your head, enough to reassure the participant that you are listening if there is more to say, but not to the point of awkward silence.

- Use follow-up questions to encourage dialogue, such as:
  - "That's interesting what you say about *xyz*, can you tell me more?"
  - "Can you explain to me what you mean by *xyz*?"
  - "You told me about *xyz*, but is that all there is? Would there be anything else?"
  - "From what I understand, for you it is *xyz* (using different words), is that right?"

- Feel free to repeat the participant's answers to make sure you understand.

Date of the interview:

DD	MM	YYYY

Location of the interview:

Interviewer

ID number: | | | | | O | H | M | - | | |

Participant

Survey ID number: | | | | | O | H | M | - | | |

Sex:

Age :

Mother tongue:

Family situation:

# individuals in household:

Participant activity:

- ☐ Agriculture, gardening
- ☐ Animal husbandry
- ☐ Hunting, fishing
- ☐ Picking and gathering (foraging)
- ☐ Employee, professional activity
- ☐ Homemaker
- ☐ Studies
- ☐ None
- ☐ Other (specify):

Main place of residence:

- ☐ Agglomerated population zone/neighborhood. Details: ____________
- ☐ Suburban area/neighborhood. Details: ____________
- ☐ Isolated aread. Details: ____________
- ☐ Other (specify): ____________

Do you prepare meals at home?

- ☐ Never
- ☐ From time to time
- ☐ Very frequently/all the time

In general, would you say your health is:

- ☐ Excellent
- ☐ Very good
- ☐ Good
- ☐ Passable
- ☐ Poor

Questionnaire on Food Systems Under Influence

Survey ID number:

Etic section : 24h Recall - Questionnaire on Dietary Diversity (QDD)

Q1. Please indicate what you ate and drank yesterday (meals and snacks), whether during the day or at night, at home or outside. Start with the first food or drink consumed in the morning. (*Note the foods and beverages mentioned in grid 1.*)

Q1a. If a cooked dish is mentioned, ask for the list of ingredients.

Q1b. Check if the person interviewed has not missed a meal or snack.

Q1c. Ask if any items have been added to consumptions.

When the person interviewed has finished their description, check the food groups consumed in grid 2

Q2. Please confirm if you ate any food belonging to (*food groups not mentioned*). (*Use the examples in grid 2 and check the associated food groups, if applicable* .)

Q3. Was yesterday a holiday or celebration where you ate special foods, or did you eat more or less than usual?

Q4. What was the origin [*sources of supply*] of the food/dishes consumed? (*See the code box [1] in grid 1* .)

Q5. What state [*degree of processing*] was the food/dish in when it was prepared for consumption? (*See the code box [2] in grid 1.*)

Q6. For the most important meal, do you consider the foods included to be: a) local or not, b) traditional or not, c) healthy or not? (*See the code box [3] in grid 1.*)

Questionnaire on Food Systems Under Influence

Survey ID number:

Emique section: Food system representations

Q7. What does eating well mean to you?

Q8. What is eating poorly?

Q9. What allows you to eat well?

Q10. What prevents you?

Q11a. Has your diet changed in your lifetime?

Q11b Why? How?

Q12a. Has [the founding event of OHM(i)] changed your diet?

Q12b Why? How?

Q13a. Do you or any household members have diet-related health problems?

Q13b Which? Where does it come from?

Q14a. What can make you sick in your diet?

Q14b Why? Examples ?

Q15a. How do you prefer to obtain your groceries?

Q15b Why? Do you have any examples of foods depending on location?

Q16. Is there anything else you would like to add?

Grid 1. Grid for recording food, drinks and meals consumed in a 24-hour period

Survey ID number:

Ante meridiem	Snack	Meridiem	Snack	Post meridiem	Snack

Codes

[1] Sources of supply:

1 = Self-acquisition (e.g.: gathering, hunting, fishing); **2** = self-production (e.g. agriculture, livestock); **3** = Borrowing, barter, exchange for work, gift from friends or relatives; **4** = Purchase - supermarket; **5** = Purchase - local market; **6** = Purchase - local business (e.g.: bakery); **7** = Online purchase; **8** = Food aid; **9** = Other (e.g.: Co-op)

[2] Degree of transformation (NOVA classification):

A = Unprocessed or minimally processed; **B** = culinary; **C** = processed; **D** = ultra-processed

[3] Perception of local, traditional and healthy:

L = local; **NL** = non-local; **T** = traditional; **NT** = non-traditional; **S** = healthy; **MS** = unhealthy

Grid 2. Grid for recording food groups consumed

Survey ID number:

**Group n°**	**Food group**	**Examples**	**YES= 1<br>NO = 0**
1	CEREALS	corn/maize, rice, wheat, sorghum, millet or any other grains or foods made from these (e.g. bread, noodles, porridge or other grain products) + *insert local foods*	
2	STARCHY ROOTS/TUBERS/OTHERS	potatoes, yams, cassava, sweet potatoes or other root foods. Also include starchy fruits such as breadfruit and plantains + *insert local foods*	
3	DARK GREEN LEAFY VEGETABLES	dark green leafy vegetables, including wild forms + *insert local foods*	
4	OTHER VEGETABLES AND UNRIPE FRUITS	other vegetables (e.g. tomato, onion, eggplant, pumpkin, carrot, squash, peppers), unripe fruits (green mangoes and green papayas) + *insert local foods*	
5	RIPE FRUITS (FRESH OR DRIED)	ripe or dried fruits (e.g. mango, melon, apricot, papaya, peach), including wild fruits and pure juices obtained from these fruits + *insert local foods*	
6	ORGAN MEAT	liver, kidney, heart or other organ meats or blood-based foods + *insert local foods*	
7	MEAT (MUSCLE)	beef, pork, lamb, goat, rabbit, game, chicken, duck, other birds, insects, reptiles, amphibiens, including wild game + *insert local foods*	
8	PROCESSED MEAT	sausages, pepperettes, ham, pâté, terrine, canned meat + *insert local foods*	
9	EGGS	eggs from chicken, duck, guinea fowl or any other egg + *insert local foods*	
10	FISH AND SEAFOOD	fresh or dried fish, molluscs (e.g., mussels, oysters, octopus), crustaceans (e.g., prawns, crabs), echinoderms (e.g., sea urchins, starfish and sea cucumbers) + *insert local foods*	
11	PULSES	dried beans, dried peas, lentils, or foods made from them (hummus or tofu, for example) + *insert local foods*	
12	OLEAGINOUS FRUITS AND SEEDS	nuts (almond, cashew, Brazil nut), oleaginous fruits (olive, avocado, and palm fruits), various seeds (peanut, sunflower and hemp seeds) or foods made from them (nut butters, tahini, "milks" made from nuts) + *insert local foods*	
13	MILK AND DAIRY PRODUCTS	milk, cheese, yogurt or other milk products + *insert local foods*	
14	OILS AND FATS	oil, fats or butter added to food or used for cooking	
15	SWEETS	sugar, honey, sweetened soda or sweetened juice drinks, sugary foods such as chocolates, candies, cookies and cakes + *insert local foods*	
16	SALTY AND FRIED FOODS	Fries (potato, sweet potato, cassava), fried dough (bokit, acras), chips (potato, sweet potato, cassava) + *insert local foods*	
17	SPICES, CONDIMENTS, DRINKS	spices (black pepper, salt), condiments (soy sauce, hot sauce), coffee, tea + *insert local foods*	
18	ALCOHOLIC BEVERAGES	beer, wine, cider and spirits + *insert local foods*	
At the individual level	Did you eat (meal or snack) OUTSIDE THE HOME yesterday?		
At the individual level	Was yesterday a holiday or celebration where you ate special foods, or did you eat more or less than usual?		
